# Fitness costs and persistence of plasmid-mediated cephalosporin resistance in *Escherichia coli*: an integrative review

**DOI:** 10.3389/fmicb.2026.1783087

**Published:** 2026-04-15

**Authors:** Lázaro López, Pamela Cangui, Denyss Guilcazo, Antonio Machado, Zachary D. Blount, Gabriel Trueba

**Affiliations:** 1 Instituto de Microbiología, Colegio de Ciencias Biológicas y Ambientales, Universidad San Francisco de Quito, Quito, Ecuador; 2 Departamento de Biologia, Faculdade de Ciências e Tecnologia, Universidade dos Açores, Ponta Delgada, Portugal; 3 CIBIO, Centro de Investigação em Biodiversidade e Recursos Genéticos, InBIO Laboratório Associado, Pólo dos Açores, Universidade dos Açores, Ponta Delgada, Portugal; 4 Department of Microbiology, Genetics, and Immunology, Michigan State University, East Lansing, MI, United States

**Keywords:** antimicrobial resistance, competition assays, *Escherichia coli*, extended-spectrum β-lactamases, fitness cost, horizontal gene transfer, One Health, plasmid

## Abstract

The global spread of resistance to third-generation cephalosporins (TGCs) in *Escherichia coli* limits therapeutic options and poses major challenges for human, animal, and environmental health. The spread of resistance genes, including those for extended-spectrum β-lactamases (ESBLs), AmpC-type β-lactamases, and carbapenemases, has been facilitated by horizontal gene transfer (HGT), often via conjugative plasmids. This plasmid-mediated mobilization has enabled rapid adaptation to front-line antibiotics across diverse bacterial populations and ecological niches. Here, we bring together an integrative synthesis of molecular mechanisms, genetic vehicles, and ecological dynamics of cephalosporin resistance in *E. coli*, alongside a PRISMA-guided quantitative synthesis of 40 studies that provide data on the fitness consequences of resistance plasmids. We have analyzed a total of 154 experimental observations to identify patterns related to plasmid host background, resistance gene family, and fitness-assay framework. Because multiple observations were frequently contributed by the same study, we accounted for hierarchical structure using mixed-effects models with Study_ID as a random intercept and evaluated key patterns in the full dataset and stratified by assay type (growth curves vs. head-to-head competition assays). Moreover, we found that fitness estimates were sensitive to assay type. For instance, head-to-head competition experiments captured a broader range of deviations from neutrality than growth curve assays, although the apparent difference in mean standardized fitness between assay types was attenuated after accounting for study-level clustering. Across the curated dataset, host-associated and resistance gene-family-associated signals were method-dependent: both were evident overall and in head-to-head competition assays, but were not retained in growth-curve-only subsets. Our analysis supports a context-dependent interpretation in which plasmid-host compatibility, resistance-gene context, ecological setting, and the measurement framework jointly shape the observed fitness consequences and dissemination potential of resistance plasmids across environments.

## 1 Introduction

Antimicrobial resistance (AMR) is a serious threat to global health. The rising prevalence of multidrug-resistant bacteria is limiting therapeutic options and compromising the effectiveness of routine medical procedures (Salam et al., 2023; Oliveira et al., 2024). A particularly concerning aspect of AMR is the widespread emergence of *Escherichia coli* strains that are resistant to third-generation cephalosporins (TGCs), which have been critical for treating serious Gram-negative infections (Galgano et al., 2025). This resistance can be conferred by many genes, including ones that encode extended-spectrum β-lactamases (ESBLs), AmpC β-lactamases, and carbapenemases, the spread of which has been facilitated by mobile genetic elements, especially conjugative plasmids (El Salabi et al., 2013).

*E. coli* is a versatile bacterium that cycles between multiple niches and can be both a commensal and a pathogen. This ecological breadth, together with its ubiquity, genetic plasticity, and capacity to acquire foreign DNA, makes *E. coli* an efficient vehicle for disseminating resistance genes (Braz et al., 2020; Naidoo and Zishiri, 2025). Moreover, *E. coli*-associated plasmids that carry genes encoding β-lactamases and other resistance determinants are common in both community and hospital settings. Emblematic of the broader AMR crisis, these plasmids are also widespread in animal and environmental reservoirs (El Salabi et al., 2013; Shaikh et al., 2014; Kim et al., 2025; Muteeb et al., 2025).

Plasmids are often costly for their hosts (Lopatkin et al., 2017; Wein et al., 2019; Alonso-Del Valle et al., 2021; Fernández-Calvet et al., 2023; López et al., 2025a). Both plasmid maintenance and the expression of plasmid-encoded genes carry physiological and fitness costs, which can reduce a host’s growth rate, competitiveness, and capacity to survive stress (Hall et al., 2021; Hofer, 2022). However, the magnitude and consequences of these fitness costs vary depending on the plasmid, the genes it carries, the genetic background of the bacterial host, and the environment in which it resides (San Millan and MacLean, 2017; Ahmad et al., 2023; Liu et al., 2024). Over time, co-adaptation between the host and the plasmid can reduce these costs, thereby allowing plasmids to persist even in the absence of selective pressure (Zwanzig et al., 2019; Yang et al., 2020; Rebelo et al., 2023; Liu et al., 2024).

Despite decades of research on plasmid-mediated antimicrobial resistance, our understanding of how these elements persist and spread remains incomplete. Molecular studies have uncovered the genetic basis of β-lactamase production (Hussain et al., 2021; Naas et al., 2023; Gross et al., 2024), and ecological surveys have revealed the ubiquity of resistant *E. coli* in human, animal, and environmental niches (Rousham et al., 2018; Pandey et al., 2024; Karabasil et al., 2025; Xu et al., 2025). Despite these advances, we lack a comprehensive framework that integrates molecular mechanisms with ecological dynamics and evolutionary outcomes. Without such a framework, it is difficult to explain why certain plasmids establish themselves successfully, much less to anticipate the conditions under which TGC resistance dissemination will continue.

Addressing this gap requires particular attention to the fitness consequences of plasmid carriage. Fitness effects, be they costs or benefits, determine whether plasmids are transient passengers or long-term residents of their host populations (Carroll and Wong, 2018; MacLean and Millan, 2019). Although many experimental studies have measured these effects (Petersen et al., 2009; Humphrey et al., 2012; Fuzi, 2016; Ma et al., 2018; Hall et al., 2021; Fernández-Calvet et al., 2023; Helekal et al., 2023; Liu et al., 2024; Pal and Andersson, 2024; Wright et al., 2024; Elg et al., 2025; López et al., 2025a), the results remain scattered and highly context-dependent, which limits broad interpretation. Developing a clear picture of the conditions that favor persistence requires synthesizing not only this evidence, but that from molecular and ecological studies.

This review aims to: (i) synthesize molecular and ecological insights into TGC resistance in *E. coli*; (ii) analyze empirical data on fitness effects through a systematic review with quantitative synthesis; and (iii) highlight key factors influencing the persistence and dissemination of resistance plasmids. We will thus integrate narrative and quantitative approaches to provide a comprehensive understanding of host–plasmid interactions in the context of antimicrobial resistance.

## 2 Molecular and evolutionary determinants of plasmid-mediated third-generation cephalosporin resistance in *Escherichia coli*

Third-generation cephalosporin (TGC) resistance in *E. coli* is largely disseminated by plasmids carrying β-lactamase genes, including ESBLs, plasmid-mediated AmpC enzymes, and carbapenemases. Although β-lactamases are classically grouped into Ambler classes A–D, their enzymatic taxonomy alone is insufficient to explain plasmid persistence. At population scale, persistence is more strongly shaped by plasmid-level features: backbone architecture, copy-number control, transfer and stability modules, associated cargo, and compatibility with the recipient host genome. These determinants influence the net fitness effect of carriage and therefore whether resistant lineages expand, stabilize, or decline over time (San Millan and MacLean, 2017; Carroll and Wong, 2018; San Millan, 2018).

Copy-number regulation is another central mechanism connecting molecular architecture to fitness. Alterations in replication control can increase resistance expression but may simultaneously elevate energetic cost and instability. Conversely, finely tuned copy-number systems can buffer burden and favor persistence. Recent evidence highlights copy-number plasticity as a key axis of adaptive trajectories in resistance plasmids, helping explain why apparently similar gene contents can yield different fitness outcomes across hosts (Ramiro-Martínez et al., 2025).

Genetic context further modulates outcomes. The same β-lactamase gene may generate different phenotypes depending on promoter strength, insertion sequence environment (e.g., ISEcp1-associated mobilization), co-localized resistance/virulence loci, and plasmid backbone. This context dependence is especially relevant for IncF lineages in *E. coli*, where host adaptation, maintenance systems (e.g., toxin–antitoxin modules), and reduced regulatory conflict are consistent with lower average carriage costs in many backgrounds. By contrast, broad-host-range or recently acquired plasmids may impose stronger short-term burdens until compensatory adjustments emerge (Carroll and Wong, 2018; Palomino et al., 2022).

Ecological setting is also important for interpreting fitness. *In vitro* monoculture assays can underestimate subtle burdens that become detectable in direct competition, while host-associated environments and structured communities can alter selective gradients and retention dynamics (Buckner et al., 2018; Zhang P. et al., 2021). Thus, measurable acquisition costs can coexist with long-term persistence when fluctuating selection and compensatory adaptation are present.

Together, these findings support an evolutionary interpretation in which initial plasmid-associated costs do not necessarily determine long-term persistence. The persistence of resistance is determined by the interaction between plasmid architecture, host background, compensatory trajectories, and environment, which provides the conceptual basis for the quantitative analyses presented below. This framework motivated the variables prioritized in our quantitative synthesis.

## 3 Fitness costs and adaptation

The evolutionary success of plasmids carrying antibiotic resistance genes in *E. coli* hinges on how they affect bacterial fitness across multiple environmental and ecological contexts (Svara and Rankin, 2011). Although these plasmids confer advantages when under selective pressure from antibiotics, they can also impose metabolic and physiological burdens on their hosts (Jian et al., 2021; Dewan and Uecker, 2023). It has been traditionally assumed that plasmid costs drive the loss of plasmids in the absence of selective agents to which they provide resistance. This assumption has been challenged by new results. In the following, we will review the empirical evidence and theoretical frameworks underlying the concept of plasmid fitness cost, how costs have been quantified, and the adaptive mechanisms that permit plasmids to persist.

### 3.1 Fitness costs of plasmid carriage

Plasmids can impose costs on their hosts a number of ways. Metabolic burdens that reduce host growth rates can arise from the energy required to replicate plasmids or to express plasmid-borne genes (Silva et al., 2012; Curtsinger et al., 2024). Plasmid-encoded promoters and regulatory sequences can also interfere with host transcriptional networks, alter the balance of sigma factors, and disrupt metabolic fluxes, creating global physiological imbalances (Billane et al., 2021; Elg et al., 2025). This metabolic perturbation framework is supported by recent evidence showing that plasmid-associated fitness costs frequently emerge through system-level effects on central metabolism and cellular physiology rather than through a single resistance determinant (Palomino et al., 2022; Hall et al., 2024). Both of these burdens can reduce the hosts’ competitiveness in mixed microbial populations (Sünderhauf et al., 2023).

Plasmids may also compromise the host’s stress response by encoding proteins that interfere with global stress tolerance regulators (e.g., RpoS), by producing membrane-associated proteins that destabilize envelope integrity, or by generating reactive oxygen species as by-products of plasmid gene expression. These effects can make plasmid-bearing bacteria more sensitive to oxidative stress, nutrient starvation, and pH fluctuations (San Millan et al., 2013; Yu et al., 2022; Li and Zhang, 2023).

Plasmid-associated fitness costs vary widely depending on plasmid size, copy number, and genetic cargo. Copy-number plasticity and replication-control tuning can also modulate the balance between resistance expression and host burden, making copy-number dynamics a central axis of plasmid adaptation (Ramiro-Martínez et al., 2025). Small, low-copy plasmids that encode only resistance determinants may impose relatively mild burdens. By contrast, large, multi-replicon plasmids with stability systems, conjugative machinery, and multiple resistance genes tend to impose higher costs, although outcomes remain host- and environment-dependent. The magnitude and nature of fitness effects -whether beneficial, neutral, or costly- can also vary with host genotype and environment (San Millan and MacLean, 2017; Carroll and Wong, 2018).

### 3.2 Experimental methods to assess fitness

The host fitness costs exerted by resident plasmids can be quantified in a number of ways that are amenable to use with *E. coli*. One of the simplest and most widely used is growth curve analysis, in which plasmid-bearing and plasmid-free strains are inoculated separately into liquid culture and their growth is monitored over time, typically via optical density or colony-forming unit counts. This approach can be used to assess effects on lag phase, exponential growth rate, and carrying capacity (Krishnamurthi et al., 2021; Mira et al., 2022; Opalek et al., 2022; Lamont et al., 2023). Because plasmid replication and the expression of plasmid-borne genes often slow growth or prolong lag time, deviations in these parameters can provide indirect indicators of plasmid-associated costs. Growth curves are straightforward, reproducible, and suitable for high-throughput comparisons, but, because strains are assayed independently, they do not capture slight differences with subtle effects on dynamics, even though they can produce large disparities in mixed populations.

Head-to-head competition assays overcome some of the drawbacks of growth curves and provide more precise measures of relative fitness. In these assays, strains that are isogenic save for the presence or absence of the plasmid are co-cultured and propagated in an experimental environment for one or more serial passages of 24 or more hours. The frequencies of the competitors are tracked over time, either by selective plating or by cell sorting using molecular markers (e.g., fluorescent proteins, etc.) (Petersen et al., 2009; Basso et al., 2020; Worthan et al., 2023; Wright et al., 2024). Assay results are typically expressed in terms of the fitness (w) of one competitor relative to that of the other, and are calculated as ratios of growth rates (Malthusian parameters) based on final population densities or changes in strain frequency (Pajon et al., 2023). Values of w below 1 indicate a fitness cost, whereas values above 1 suggest a fitness advantage (Barrick et al., 2020, 2023; Pajon et al., 2023). These metrics provide a standardized framework for comparing experiments across different plasmid–host combinations and environmental conditions. Moreover, because both strains experience identical conditions, any differences in fitness must stem from the net fitness effects of the plasmid. These competitions integrate over entire growth cycles, making them highly sensitive and capable of detecting even small fitness differences that would not be readily apparent from growth curves alone.

Transcriptomics, proteomics, metabolomics, and other molecular and omic methods provide mechanistic insights into plasmid-associated fitness effects. These methods can reveal how plasmids affect host cellular machinery by quantifying genome-wide changes in gene expression, protein levels, and metabolic fluxes. However, the mechanistic correlates illuminated by these methods are insufficient to establish causality without integration with perturbation experiments (Kocharunchitt et al., 2011; Liu et al., 2020). Plasmids may upregulate stress response pathways, alter central metabolism, and increase membrane protein expression, so this integration is critical to explaining the origin of plasmid costs.

Finally, *in vivo* models add an additional layer of ecological realism. For instance, mouse gut colonization experiments enable researchers to evaluate plasmid fitness under host-associated conditions. Model-specific host factors limit generalization across hosts and niches, underscoring the value of parallel *in vitro* and *in vivo* designs (Johnson et al., 2015; Alonso-Del Valle et al., 2021; Ott and Mellata, 2022). These models incorporate interactions with the resident microbiota, immune responses, and nutrient fluctuations that cannot be replicated *in vitro*. Consistent with this, animal and host-associated models have documented sustained plasmid persistence and transfer under *in vivo* ecological constraints, including contexts where *in vitro* assays detect measurable costs (Buckner et al., 2018; Alonso-Del Valle et al., 2021). They are particularly valuable for understanding how plasmids persist in natural populations, where selective pressures and ecological complexity may differ greatly from laboratory environments.

Together, these complementary methods—from simple growth assays to complex *in vivo* models—can provide a multi-scale view of the fitness effects of plasmid carriage. Combining them is essential to capturing both the mechanistic underpinnings and ecological consequences of plasmid-mediated resistance.

### 3.3 Compensation mechanisms

Despite their intrinsic costs to hosts, plasmids can persist over time as compensatory mechanisms evolve that reduce those costs at either the chromosomal or plasmid levels (Carroll and Wong, 2018). Chromosomal mutations are often in host regulatory and metabolic genes. For example, mutations in RNA polymerase subunits (e.g., *rpoB*, *rpoC*) can reduce the transcriptional burden of plasmid gene expression, thereby improving the growth of plasmid host cells (Brandis and Hughes, 2018; Liu et al., 2024). Similarly, alterations in global regulators (e.g., H-NS or CRP) can rebalance gene expression and mitigate plasmid costs (Curtsinger et al., 2024; Elg et al., 2025). Alternatively, plasmid streamlining involves the deletion of redundant or non-essential plasmid genes, or mutation of replication control regions, thereby causing the plasmid to evolve to have lower energetic costs (Bedhomme et al., 2017; Dewan and Uecker, 2023). Large multiresistance plasmids, for instance, often lose conjugation machinery and accessory genes during prolonged maintenance in stable laboratory environments, thereby becoming “leaner” and less costly while retaining key resistance functions. Finally, compensation can arise via regulatory tuning of plasmid gene function through the accumulation of mutations in promoter regions or the acquisition of repressor genes that downregulate the expression of costly genes, while resistance determinants remain intact (Hall et al., 2021; Elg et al., 2025). A well-studied example is the downregulation of costly conjugation operons, which reduces fitness costs without compromising resistance.

Finally, host–plasmid coevolution during extended associations can result in the stable integration of plasmid functions into host physiology (De Gelder et al., 2008; Brockhurst and Harrison, 2022; Li et al., 2023). This coevolution can occur quickly. In laboratory experiments, resistance plasmid-carrying *E. coli* with no detectable plasmid-associated costs have evolved on the order of hundreds of generations owing to compensatory chromosomal and plasmid mutations. Indeed, in some experiments, plasmids have evolved mutualistic interactions with their hosts.

Together, these mechanisms illustrate the dynamic nature of host–plasmid relationships. Rather than static burdens, plasmids and their bacterial hosts continuously adapt to one another, thereby allowing resistance plasmids to persist and spread even in the absence of direct antibiotic selection.

### 3.4 Genetic interdependence and epistatic plasmid-host interactions

Beyond direct compensatory adaptations, the persistence of plasmids is deeply influenced by epistatic interactions (Liu et al., 2024), i.e., non-additive genetic relationships between plasmid and chromosomal loci that shape how each evolves in the context of the other. Epistasis determines whether the phenotypic effect of a mutation depends on the genetic background in which it occurs, and in plasmid-bearing bacteria this creates a complex web of dependencies between two coexisting genomes. Rather than acting as autonomous elements, plasmids integrate into host regulatory and metabolic networks. Their replication, transcriptional control, and maintenance functions interact with host pathways in ways that can either stabilize or destabilize the association. These interactions generate idiosyncratic fitness landscapes: a chromosomal mutation that is beneficial in one plasmid background may be neutral or even deleterious in another, and conversely, a plasmid mutation that alleviates costs in one host genotype may increase them in a different one (Billane et al., 2021; DelaFuente et al., 2024). Epistasis can therefore constrain or facilitate evolutionary trajectories. It can limit horizontal transfer by creating host-specific compatibility profiles, or alternatively, it can foster long-term stability through reciprocal adaptation. In some cases, epistasis even enables the emergence of mutualistic dynamics, where the presence of the plasmid becomes essential to the optimal functioning of the host regulatory network (Orlek et al., 2017). Understanding plasmid–host evolution through the lens of epistasis highlights that adaptation is multidimensional: every genetic change reshapes the selective environment of the other genome.

### 3.5 Environmental and ecological contexts

Plasmid fitness effects can vary dramatically across environments and hosts (Melbinger and Vergassola, 2015). In nutrient-rich, antibiotic-free environments, for instance, fitness costs are often more detectable because resource abundance and intense competition can magnify even small differences in growth rate or efficiency (Ma et al., 2018; López et al., 2025a). Under these conditions, plasmid-free strains often outcompete plasmid-bearing strains in the short-term, particularly before compensatory adaptation or ecological buffering occurs. By contrast, spatially structured communities, as found in biofilms, can lead to mitigated plasmid costs by reducing direct competition between individual cells, and allowing plasmid-carrying cells to persist despite lower growth rates (Metzger et al., 2022). Moreover, plasmids can provide cooperative benefits, like β-lactamase secretion, that protect neighboring cells and spread the benefit of resistance across the community, while offsetting individual costs. Thus, short-term competitive disadvantages do not preclude long-term persistence, because compensatory evolution, host-plasmid coadaptation, and fluctuating selection can stabilize plasmid carriage over time.

Plasmid retention can also be favored in environments in which antibiotic exposure fluctuates or persists at low levels (Jordt et al., 2020). Even if plasmids impose a measurable burden during antibiotic-free intervals, the decisive advantage their carriers possess during intervals of antibiotic presence creates a dynamic balance in which plasmids can persist in populations over long timescales regardless of short-term costs. Similarly, sub-inhibitory antibiotic concentrations can inhibit susceptible cell growth and subtly tip the competitive balance in favor of plasmid carriers.

Host genetic background can also strongly modulate the costs of identical plasmids in wildly varying ways across different *E. coli* strains (Humphrey et al., 2012; Alonso-Del Valle et al., 2021). This variability arises from differences in host tolerance for plasmids owing to variation in host regulatory networks, stress responses, and metabolic capacities. Long-term coevolution between hosts and plasmids leads to better matches between plasmids and host physiologies. Plasmids acquired from phylogenetically distant donor strains typically impose higher fitness costs than those from closely related strains because the evolutionary distance reduces the degree of plasmid-host matching (Zhang et al., 2019). By contrast, closely related hosts will have more similar physiologies, so the disparity in the degree of physiological match will be lower, and the plasmid cost with it.

## 4 Quantitative evidence synthesis

What are the fitness effects of cephalosporin-resistant plasmids in *E. coli*? To answer this question, we used PRISMA 2020 (Preferred Reporting Items for Systematic Reviews and Meta-Analyses) guidelines to conduct a systematic review with quantitative synthesis of studies addressing this question. This approach allowed us to integrate diverse experimental datasets into a coherent framework with which to evaluate the relative influence of plasmid host background and resistance.gene family context, and the method by which fitness effects were measured. The PRISMA flow diagram is provided in Supplementary Figure S1.

### 4.1 Literature search strategy

We carried out a comprehensive search in PubMed, Scopus, and Web of Science on May 27, 2024. The Boolean query used was:

*(Escherichia AND coli OR E. coli) AND (cephalosporin OR betalactamase OR β-lactamases OR cephem OR Extended-Spectrum β-Lactamases OR ESBL) AND fitness.*

Titles and abstracts were screened against predefined eligibility criteria, and duplicates were removed using Zotero. The study-selection workflow is shown in Supplementary Figure S1.

We included papers that reported original research that included quantitative data on the fitness of *E. coli* strains harboring plasmids that confer resistance to cephalosporins. To ensure meaningful comparisons, we only considered studies that analyzed both plasmid-bearing strains and their isogenic plasmid-free counterparts, and which explicitly reported the β-lactamase gene(s) and the plasmid source. We excluded non-original publications (e.g., reviews), non-English publications, and studies that did not provide clear descriptions of the experimental conditions and the methods used to assess bacterial fitness. The complete list of included studies is provided in Supplementary Table S1.

### 4.2 Data extraction and standardization

For each eligible study, we extracted observation-level data on plasmid host background, recipient-strain background, resistance gene identity, fitness-assay type, and the original fitness metric reported by the source study. Because source studies reported heterogeneous fitness metrics -including relative fitness, relative growth rate, and selection-derived measures- we harmonized these values onto a comparative scale centered on w = 1, where values below 1 indicate reduced fitness relative to the plasmid-free comparator and values above 1 indicate improved relative performance. Directly reported relative fitness and relative growth values were retained when already expressed on this scale. Selection-derived measures were converted only when the study-specific definition and competition design permitted a defensible transformation consistent with the reported experimental framework. Observations based on direct competition-index CFU-ratio formulations that could not be defensibly standardized to the comparative fitness scale without introducing arbitrary assumptions were excluded from the quantitative synthesis. Following this metric-curation step, the final standardized quantitative dataset comprised 40 studies contributing 154 observation-level fitness records. To ensure transparency, Supplementary Data S1 provides the curated observation-level dataset together with the original metric label, original metric definition, standardization rule applied, standardization formula, and study-specific notes. These notes explicitly document the rationale and transformation approach used for each retained observation, including cases in which metric interpretation required study-specific clarification. This curation step was performed before model fitting, and only the harmonized dataset was used for inferential analyses.

### 4.3 Exploratory data visualization

We generated exploratory visualizations to examine fitness-value distributions across assay type, plasmid host bacterium, and resistance-gene identity/family, including a horizontal reference line at *w* = 1.0. These plots were used as descriptive summaries and were interpreted in conjunction with mixed-effects models that account for study-level clustering.

### 4.4 Statistical analysis

All analyses were performed in R (v4.5.1). The primary quantitative outcome was the standardized comparative fitness value (w): Fitness_value_standardized_w. Because multiple observations were frequently contributed by the same source study (i.e., several observations shared a common Study_ID), we did not treat all observations as statistically independent in the main inferential analyses. Instead, we used linear mixed-effects models with Study_ID specified as a random intercept to account for within-study clustering and to avoid inflating the effective sample size.

Analyses were organized into three datasets: (i) the full dataset including all fitness-assay types, (ii) a growth-curve-only subset, and (iii) a head-to-head-competition-only subset. We used these stratified analyses to evaluate whether biological signals were method-dependent (i.e., whether patterns detected in the full dataset were retained within each assay framework).

For each analysis, we fitted mixed-effects models of the form Fitness_value_standardized_w ~ predictor + (1 | Study_ID), where the predictor corresponded to: (i) fitness-assay type (growth curves vs. head-to-head competition), (ii) plasmid host bacterium, or (iii) resistance gene family group (as defined in the curated dataset). Overall fixed-effect significance was assessed using Type III ANOVA. Where appropriate, estimated marginal means (EMMs) and 95% confidence intervals were obtained using the emmeans package to summarize model-adjusted group means. We also quantified the degree of within-study clustering by calculating the intraclass correlation coefficient (ICC) from the fitted mixed-effects model. Because subgroup sizes were often imbalanced and some categories were sparse (especially after assay stratification), pairwise contrasts were interpreted as exploratory rather than confirmatory. Variant-level analyses were not used for primary inference; instead, they were retained as descriptive, hypothesis-generating summaries and are presented in Supplementary Figure S2.

### 4.5 Integrated findings and interpretation

The study-selection process is summarized in Supplementary Figure S1. A total of 45 studies met the inclusion criteria for the qualitative/integrative review. Following observation-level metric curation for the standardized quantitative synthesis, 5 studies were excluded because their reported fitness metrics could not be defensibly harmonized to the comparative fitness scale (w) without introducing arbitrary assumptions. The final standardized quantitative dataset therefore comprised 40 studies contributing 154 observation-level fitness records (Supplementary Table S1; Supplementary Data S1).

#### 4.5.1 Measurement method shapes the apparent detection of fitness effects

**Figure 1:**
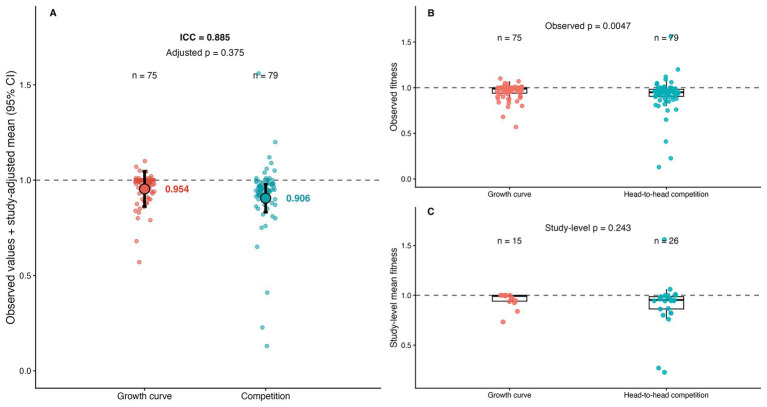
Fitness estimates by measurement method before and after accounting for study-level clustering. Panel **(A)** shows the mixed-effects model summary comparing standardized fitness values across growth-curve and head-to-head competition assays, with model-adjusted estimated means and 95% confidence intervals; the adjusted effect of assay type was not statistically significant after accounting for Study_ID as a random intercept (*p* = 0.368; ICC ≈ 0.885). Panel **(B)** shows the unadjusted observation-level distribution of standardized fitness values by assay type. Panel **(C)** shows a study-level sensitivity summary based on mean fitness values aggregated within studies. In all panels, the dashed horizontal line indicates neutral fitness (*w* = 1).

In unadjusted observation-level comparisons, standardized fitness values differed between growth-curve and head-to-head competition assays, with head-to-head competition showing a broader distribution of deviations from neutrality than growth-curve measurements. However, because multiple observations were frequently contributed by the same study, we evaluated this pattern using a linear mixed-effects model with Study_ID as a random intercept. After accounting for study-level clustering, the effect of assay type was attenuated and was no longer statistically significant (mixed-effects model, *p* = 0.368; Figure 1). The intraclass correlation coefficient (ICC ≈ 0.885) indicated substantial within-study dependence, showing that much of the apparent raw variation was structured by study of origin rather than by assay type alone.

Despite this attenuation in the adjusted model, the descriptive distributions remained informative: growth-curve measurements were more tightly clustered around neutrality (*w* ≈ 1), whereas head-to-head competition assays captured a broader range of both fitness costs and fitness gains. This descriptive contrast is consistent with the idea that competition-based designs may better reveal context-dependent deviations from neutrality than monoculture-based measurements. At the same time, our reanalysis shows that unadjusted observation-level contrasts can overstate evidence when study-level clustering is not taken into account.

#### 4.5.2 Plasmid host background is method-dependent in its association with fitness outcomes

**Figure 2:**
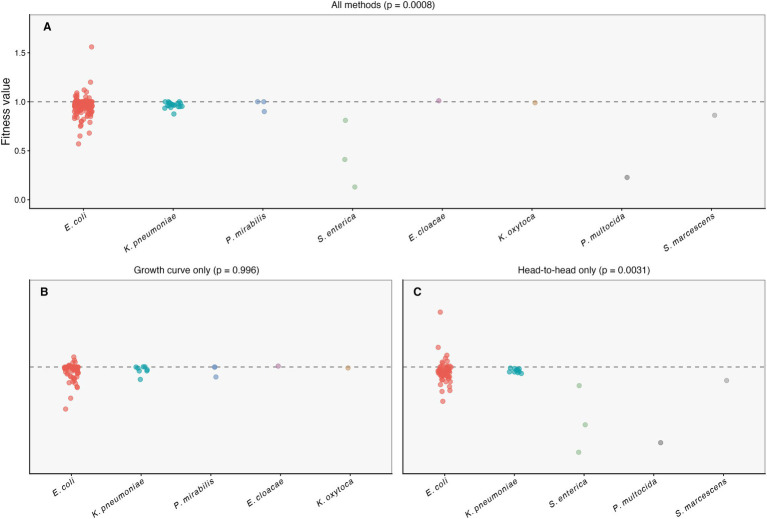
Fitness by plasmid host bacterium across assay frameworks. Standardized fitness values (*w*) are shown by plasmid host bacterium for **(A)** all observations (all assay types), **(B)** growth-curve-only observations, and **(C)** head-to-head-competition-only observations. Points represent individual observations; the dashed line indicates neutral fitness (*w* = 1). Panel titles report the mixed-effects model *p*-values for Host_Full (with Study_ID as a random intercept). Because subgroup sizes are imbalanced and several host categories have sparse representation, species-specific contrasts are interpreted as exploratory.

Across the standardized quantitative dataset (154 observations), plasmid host bacterium was associated with variation in standardized fitness values; however, subgroup sizes were highly unbalanced, and several host categories were represented by very small n. To account for within-study clustering, we evaluated this association using mixed-effects models with Study_ID as a random intercept. In the full dataset (all assay types), plasmid host bacterium was significantly associated with fitness variation (mixed-effects model, *p* = 0.0008; Figure 2A). When stratified by assay framework, this host-associated signal was not retained in the growth-curve-only subset (*p* = 0.996; Figure 2B) but remained evident in the head-to-head-competition-only subset (*p* = 0.0031; Figure 2C). These results indicate that the visibility of host-associated fitness differences is method-dependent and is primarily captured in head-to-head competition assays, while growth-curve measurements showed comparatively limited separation across host categories.

Given the imbalance of subgroup sizes and sparse categories (including groups with *n* = 1–3), species-specific contrasts should be interpreted as exploratory. Nevertheless, the overall pattern is consistent with the hypothesis that plasmid–host compatibility may differ between native and non-native host backgrounds, with potential consequences for the magnitude of fitness perturbations observed in competition-based assays.

#### 4.5.3 Resistance gene-family signals are method-dependent

**Figure 3:**
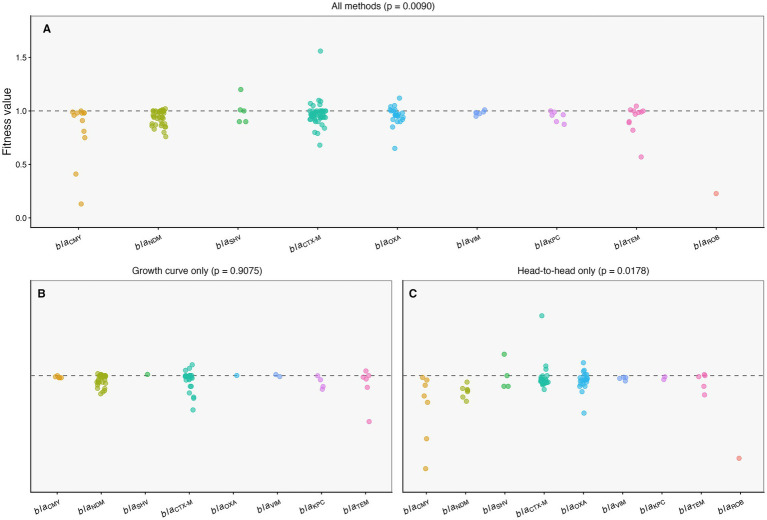
Fitness by resistance gene family across assay frameworks. Standardized fitness values (*w*) are shown by curated resistance gene family group (Gene_Family_Main) for **(A)** all observations (all assay types), **(B)** growth-curve-only observations, and **(C)** head-to-head-competition-only observations. Points represent individual observations; the dashed line indicates neutral fitness (*w* = 1). Panel titles report the mixed-effects model *p*-values for Gene_Family_Main (with Study_ID as a random intercept). Because subgroup sizes are imbalanced and several gene-family categories are sparsely represented, family-level contrasts are interpreted as exploratory.

Because resistance-gene annotations in source studies were heterogeneous (including mixed labels, combined gene sets, and inconsistent reporting of families versus variants), we defined a curated main resistance gene-family variable (Gene_Family_Main) for standardized quantitative synthesis (Supplementary Data S1). Using mixed-effects models with Study_ID as a random intercept, resistance gene family was associated with variation in standardized fitness values in the full dataset (mixed-effects model, *p* = 0.0090; Figure 3A). When stratified by assay framework, this gene-family signal was not retained in the growth-curve-only subset (*p* = 0.9075; Figure 3B) but remained evident in the head-to-head-competition-only subset (*p* = 0.0178; Figure 3C). Thus, as with plasmid host background, the visibility of gene-family-associated fitness differences was method-dependent and was primarily captured in head-to-head competition assays.

Given the imbalance of subgroup sizes across gene families and sparse representation for some categories, these patterns should be interpreted as exploratory at the subgroup level. Nevertheless, the overall results indicate that gene-family context can contribute to fitness variation in competition-based assays, while growth-curve measurements show limited separation across families.

#### 4.5.4 Additional exploratory analyses and overall synthesis

We recorded additional variables when reported by source studies, including recipient-strain origin (laboratory vs. wild-type *E. coli*) and resistance gene variant annotations. However, these variables were inconsistently reported across studies and frequently confounded with study-specific design features; in addition, variant categories were often sparse and became further fragmented after stratification by assay type. We therefore retained recipient-origin patterns and variant-level summaries as descriptive, hypothesis-generating context rather than as a basis for primary inference (Supplementary Figure S2).

On balance, our reanalysis supports a context-dependent interpretation in which plasmid-host compatibility, resistance-gene context, and experimental measurement framework jointly shape observed fitness outcomes. Accounting for study-level clustering substantially reduced the apparent evidence for assay-type differences in mean fitness, highlighting the importance of hierarchical structure in this literature. At the same time, head-to-head competition assays captured a broader range of deviations from neutrality than growth-curve assays in the raw distributions. Consistent with this method dependence, both plasmid host background and resistance gene-family group were associated with standardized fitness variation overall and within head-to-head competition assays, but these signals were not retained in growth-curve-only subsets. Together, these findings emphasize that robust inference about plasmid fitness requires transparent metric harmonization and analytical approaches that account for study-level clustering.

## 5 Epidemiology and ecological implications

Understanding the dissemination and persistence of plasmid-mediated cephalosporin resistance in *E. coli* requires contextualizing fitness dynamics within clinical, veterinary, and environmental sectors. Plasmid host backgrounds, genetic content, and host-plasmid compatibility are shaped by selective pressures that vary across clinical, veterinary, and environmental settings. In this section, we synthesize contextual evidence from the broader literature to examine how plasmid-driven resistance manifests and evolves across these settings; this section therefore does not derive directly from our quantitative dataset. Our aim here is to situate our findings within clinical, veterinary, and environmental sectors relevant to One Health.

### 5.1 Clinical settings

In hospitals and outpatient settings, selective pressure from intensive antibiotic use drives the enrichment of *E. coli* strains harboring resistance plasmids, particularly those encoding ESBLs and carbapenemases. Among clinical isolates, *bla*_CTX-M_ genes—especially *bla*_CTX-M-15_—dominate and are frequently found on IncF plasmids in pandemic clones such as *E. coli* ST131 (Banerjee and Johnson, 2014; Nicolas-Chanoine et al., 2014). These strains exhibit high transmissibility and are often multidrug-resistant, combining resistance to fluoroquinolones, aminoglycosides, and sulfonamides (Fursova et al., 2022; Rana et al., 2022).

The hospital environment also promotes the co-selection of resistance determinants owing to co-resistance and cross-resistance mechanisms (Goryluk-Salmonowicz and Popowska, 2022). Dense microbial populations and biofilm formation in medical devices (e.g., catheters) also facilitate horizontal gene transfer by allowing plasmid exchange between pathogens and commensals (Sheikh et al., 2025).

### 5.2 Veterinary and agricultural sources

Livestock production systems are major reservoirs of resistant *E. coli* strains, because of extensive use of prophylactic and growth-promoting antibiotics (Murray et al., 2024). Studies have shown that *bla*_CMY_- and *bla*_CTX-M_-carrying plasmids are highly prevalent in poultry, swine, and cattle. These plasmids, often of the IncI1 or IncA/C group, circulate within animal microbiota and are shed into the environment via feces, contributing to the contamination of soil and water systems (Ferreira et al., 2017; Touzain et al., 2018; Zhang Y. L. et al., 2021).

Notably, *E. coli* strains from animals can harbor resistance plasmids that are structurally and functionally similar to those found in human clinical isolates, supporting the hypothesis of zoonotic transmission (Lyimo and Sonola, 2025). Farm workers, food handlers, and consumers may be exposed to these strains through both direct contact or ingestion of undercooked animal products (Karwowska, 2024).

### 5.3 Environmental reservoirs

Environmental compartments, including surface waters, sediments, sewage, and agricultural runoff, each represent important sources of resistance genes. Wastewater treatment plants (WWTPs) receive inputs from hospitals, households, and agricultural operations, making them convergence points for diverse microbial communities and mobile genetic elements (Fouz et al., 2020; Drane et al., 2024).

While WWTPs reduce total bacterial loads, they may not fully eliminate resistant bacteria or extracellular DNA. Indeed, sub-inhibitory concentrations of antibiotics in effluents can select for plasmid-carrying strains (La Rosa et al., 2025). Studies have shown that resistance plasmids recovered from environmental samples include IncP, IncN, and IncQ types, which are known for their broad host range and high mobility (Karkman et al., 2018; Mutuku et al., 2022).

In natural ecosystems, bacteria carrying resistance plasmids may act as gene reservoirs that maintain and spread resistance even in the absence of anthropogenic antibiotics. Wildlife—including birds, rodents, and insects—can act as vectors of these strains, thereby facilitating the dispersal of resistant *E. coli* across large geographical areas.

### 5.4 Evolutionary compatibility and persistence

Our quantitative synthesis supports the idea that plasmid-host compatibility may influence long-term maintenance. In the curated dataset, host-associated fitness signals were evident in the full analysis and in head-to-head competition assays, but were not retained in growth-curve-only analyses. This pattern suggests that associations between plasmid host background and fitness outcome are method-dependent and are more readily detected in competition-based designs.

At the same time, host categories were highly imbalanced, and several non-*E. coli* groups were represented by very small numbers of observations. Therefore, host-category contrasts should be interpreted as exploratory rather than as definitive evidence for specific species-level effects. Taken together, these patterns are consistent with the broader idea that compatibility between plasmids and bacterial genetic backgrounds can shape the magnitude of observed fitness effects and, by extension, the likelihood of persistence.

Ultimately, the interplay between ecological niche, selective pressure, and host–plasmid compatibility helps determine whether resistance plasmids are maintained, lost, or stabilized over time. The recurrence of antibiotic exposure, microbial competition, and host population dynamics all likely contribute to these outcomes.

## 6 Discussion

The convergence of molecular, ecological, and evolutionary perspectives in this review highlights the complex dynamics governing the persistence and dissemination of cephalosporin-resistant plasmids in *E. coli*. By integrating quantitative fitness evidence with broader epidemiological and environmental literature, we provide a multifaceted understanding of the interplay between plasmid carriage and bacterial adaptability. In the revised quantitative synthesis, this framework is further strengthened by explicit metric curation and by inferential analyses that account for study-level clustering, while remaining clear about the inference limits imposed by dataset heterogeneity.

### 6.1 Context-dependent fitness effects

A central finding from our revised quantitative synthesis is that the apparent detection of plasmid-mediated fitness effects is strongly context-dependent and influenced by analytical framework. In unadjusted observation-level distributions, head-to-head competition assays captured a broader range of deviations from neutrality than growth-curve analyses. However, after accounting for study-level clustering with mixed-effects models, the mean difference between assay types was attenuated and was no longer statistically significant. This indicates that part of the apparent method effect in the raw data reflects the hierarchical structure of the literature, in which multiple observations are frequently contributed by the same study.

Even so, the descriptive contrast remains biologically informative. Growth-curve measurements tended to cluster more tightly around neutrality, whereas competition-based designs captured a wider spread of both fitness costs and benefits. This interpretation is consistent with prior work suggesting that subtle biological costs may be underestimated in monoculture experiments and become more visible under direct competition, where resource limitation and inter-strain interactions intensify selective pressures (Fink and Manhart, 2024; Kehila and Tokuriki, 2024; López et al., 2025a). Taken together, these results suggest that assay framework can influence the visibility of fitness signals, but that such differences should be interpreted cautiously when study-level dependence is not explicitly modeled.

### 6.2 Plasmid host background and co-adaptation

Our revised analyses indicate that plasmid host background is associated with standardized fitness variation, but that this signal is method-dependent. In the full dataset, plasmid host bacterium was associated with fitness outcomes; however, this association was not retained in growth-curve-only analyses and remained evident primarily in head-to-head competition assays. This pattern suggests that host-associated fitness differences are more readily captured in competition-based designs than in monoculture growth measurements.

At the same time, subgroup sizes across host categories were highly imbalanced, and several non-*E. coli* categories were represented by very small numbers of observations. Species-level contrasts should therefore be interpreted as exploratory. Rather than supporting deterministic claims about particular plasmid host bacterium, these data are more consistent with the broader hypothesis that plasmid-host compatibility varies across genetic backgrounds. Long-term associations between plasmids and hosts may promote partial accommodation through regulatory tuning, compensatory mutations, and reduced expression of costly traits (De Gelder et al., 2008; O’Brien and Wolf, 2017; Wedel et al., 2023). Such compatibility may contribute to stable plasmid maintenance in the absence of direct selection and may help explain the repeated success of particular plasmid-host combinations, including IncF-*bla*_CTX-M_ plasmids in *E. coli* ST131 (Nicolas-Chanoine et al., 2014; Yang et al., 2015).

### 6.3 Implications for resistance surveillance

Current AMR surveillance often emphasizes gene prevalence while underrepresenting host background and mobile-element context (Simjee et al., 2018; Oliveira et al., 2024). However, plasmids are highly heterogeneous and frequently contain multiple replicons or mosaic architectures that are not fully resolved by classical plasmid-typing frameworks (Orlek et al., 2017). Our findings support the value of integrating plasmid-resolved and whole-genome sequencing to better characterize host-plasmid associations. Field surveys increasingly show that even within localized settings, plasmid architectures are highly diverse, underscoring the need for structure-based classification frameworks (Salinas et al., 2024; Xu et al., 2024; López et al., 2025b).

### 6.4 Environmental dimensions of resistance persistence

The following subsection synthesizes contextual evidence from the broader literature and is not derived directly from our quantitative dataset. Its purpose is to place our findings within a One Health perspective, emphasizing the clinical, veterinary, and environmental interfaces through which resistance plasmids can persist and spread.

Consistent with a One Health framework, environmental reservoirs, including WWTPs, agricultural runoff, and wildlife, can act as important interfaces for the convergence of human, animal, and environment-associated microbiota (Fouz et al., 2020; Delgado-Blas et al., 2021). These reservoirs can facilitate both spread of resistance genes and recombination/evolution of new plasmid variants. Sub-inhibitory concentrations of antibiotics, heavy metals, and biocides in these environments may provide sufficient selection pressure to sustain resistance plasmids without overt clinical exposure (Chukwu et al., 2023; Drane et al., 2024). Consequently, integrating environmental surveillance with clinical AMR data could reveal hidden reservoirs and transmission routes, particularly in low-resource or agricultural settings where monitoring is less developed. Our results thus reinforce the importance of a One Health approach to resistance management.

### 6.5 Simulation-based approaches to plasmid evolution

Given the extraordinary diversity of plasmids and *E. coli* lineages (Arredondo-Alonso et al., 2025), fully deterministic prediction is unlikely to capture the complexity of plasmid-host interactions. However, the growing availability of comparative fitness data supports simulation frameworks that can explore how different factors -such as plasmid architecture, resistance gene identity and expression, host genetic background, and ecological context- shape evolutionary trajectories (Dewan and Uecker, 2023).

These *in silico* approaches are best treated as hypothesis-generating tools that can identify emergent patterns and conditions favoring plasmid persistence. Integrating such simulations with genomic and ecological data will be essential for building a mechanistic understanding of plasmid evolution and for informing resistance surveillance and containment strategies. Model outputs should be iteratively tested with experimental and field data.

### 6.6 Limitations and future directions

Our analysis is limited by heterogeneity in experimental design, metadata completeness, strain background, assay implementation, and the original fitness metrics reported across studies. Although we curated metrics to construct a standardized comparative fitness scale, not all reported formulations were directly comparable, and observations based on direct competition-index ratio metrics that could not be defensibly harmonized were excluded from the quantitative synthesis. In addition, multiple observations were frequently contributed by the same study, meaning that observation-level comparisons can overstate evidence if study-level clustering is not taken into account.

Many of the studies we screened also lacked complete metadata or did not report experiments under sufficiently comparable conditions. Long-term evolutionary studies that could provide insights into plasmid persistence beyond initial carriage effects remain relatively rare. Future studies should prioritize standardization of fitness metrics and assay conditions, explicit documentation of comparator definitions, the exploration of plasmid evolution under dynamic and ecologically realistic conditions, and the integration of genomic, transcriptomic, and phenotypic data for a systems-level understanding. Extending these efforts to mixed microbial communities and biofilm-associated settings will also be important for capturing the ecological complexity of plasmid–host interactions.

## 7 Conclusion

Plasmid-mediated resistance to third-generation cephalosporins in *E. coli* illustrates how horizontal gene transfer contributes to microbial adaptation across clinical, animal, and environmental settings. Our integrative review indicates that the persistence and dissemination of resistance plasmids are not explained solely by the presence of resistance genes, but by the interaction among plasmid-host compatibility, fitness consequences of carriage, compensatory processes, ecological context, and the experimental framework used to measure fitness.

In the revised quantitative synthesis, explicit metric curation and study-level adjustment substantially improved the interpretability of the dataset. After accounting for within-study clustering, the apparent difference in mean fitness between assay types was attenuated, but head-to-head competition assays still captured a broader range of deviations from neutrality in the raw distributions than growth-curve assays. Host-associated and resistance gene-family-associated signals were also method-dependent: both were evident overall and within head-to-head competition analyses but were not retained in growth-curve-only subsets. These findings highlight that methodological choices can influence which biological patterns become visible in comparative plasmid-fitness studies.

Overall, our results support the need to evaluate antimicrobial resistance through a multifactorial lens that includes plasmid biology, host ecology, metric transparency, and appropriate statistical treatment of hierarchical data. Addressing the global AMR crisis will require not only innovations in diagnostics and treatment, but also a deeper understanding of the evolutionary and ecological forces that shape resistance emergence and persistence. Recognizing plasmids as active evolutionary partners, rather than passive carriers of resistance, can improve our capacity to anticipate and mitigate the spread of antimicrobial resistance.
