# Supplementary material for: Fitness costs and persistence of plasmid-mediated cephalosporin resistance in Escherichia coli: an integrative review
Source: Front Microbiol. 2026 Apr 15;17:1783087. doi: 10.3389/fmicb.2026.1783087 (PMC13124721; doi:10.3389/fmicb.2026.1783087)
Supplement: Supplementary file 2 [file Presentation_1.PDF]

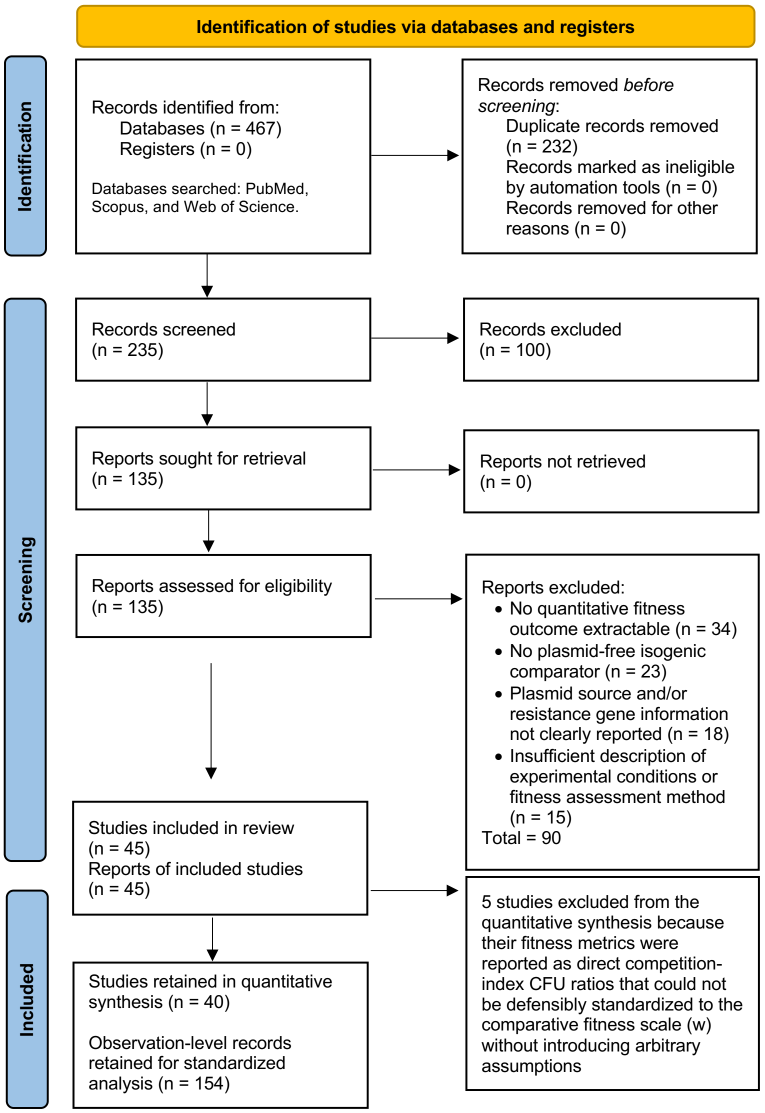

Source: Page MJ, et al. BMJ 2021;372:n71. doi: 10.1136/bmj.n71.

This work is licensed under CC BY 4.0. To view a copy of this license, visit https://creativecommons.org/licenses/by/4.0/

Supplementary Figure S1. PRISMA 2020 flow diagram of study selection and quantitative synthesis curation.

The flow diagram summarizes identification, screening, eligibility assessment, and study inclusion for this review. A total of 45 studies met the inclusion criteria for the qualitative/integrative review. Following observation-level metric curation for the quantitative synthesis, 5 studies were excluded because their fitness metrics were reported as direct competition-index CFU ratios that could not be defensibly standardized to the comparative fitness scale (w) without introducing arbitrary assumptions. The final standardized quantitative dataset therefore comprised 40 studies contributing 154 observation-level fitness records.

Supplementary Table S1. Characteristics of the 40 studies retained for the standardized quantitative synthesis.

Study-level metadata for all included publications: study ID, title, authors, DOI, publication year, continent, and country.

Study_ID	Paper Title	Authors	DOI	Year	Continent	Country
**S001**	Insertion Sequences Determine Plasmid Adaptation to New Bacterial Hosts.	Wedel, Emilia; Bernabe-Balas, Cristina; Ares-Arroyo, Manuel; Montero, Natalia; Santos-Lopez, Alfonso; Mazel, Didier; Gonzalez-Zorn, Bruno	10.1128/mbio.03158-22	2023	Europe	Spain
**S002**	Negative frequency dependent selection on plasmid carriage and low fitness costs maintain extended spectrum β-lactamases in Escherichia coli.	Dimitriu, Tatiana; Medaney, Frances; Amanatidou, Elli; Forsyth, Jessica; Ellis, Richard J.; Raymond, Ben	10.1038/s41598-019-53575-7	2019	Europe	United Kingdom
**S003**	Spreading Advantages of Coresident Plasmids bla(CTX-M)-Bearing IncFII and mcr-1-Bearing IncI2 in Escherichia coli.	He, Kun; Li, Wenya; Zhao, Bing; Xu, Hui; Pan, Yushan; He, Dandan; Hu, Gongzheng; Wu, Hua; Yuan, Li	10.1128/spectrum.01706-21	2022	Asia	China
**S004**	Characterization of IncI1/ST71 and IncF18:A-:B1 multidrug-resistance plasmids from an avian Escherichia coli isolate.	Zhang, Teng-Li; He, Dan-Dan; Liu, Ying-Ying; Yu, Li-Jie; Hu, Gong-Zheng; Pan, Yu-Shan	10.1016/j.plasmid.2022.102651	2022	Asia	China
**S005**	Genetic and Phenotypic Characterization of the Novel Metallo-β-Lactamase NDM-29 From Escherichia coli.	Zhu, Ying; Jia, Xinmiao; Jia, Peiyao; Li, Xue; Yang, Qiwen	10.3389/fmicb.2021.743981	2021	Asia	China
**S006**	Fitness effects of blaCTX-M-15-harbouring F2:A1:B- plasmids on their native Escherichia coli ST131 H30Rx hosts.	Palkovicova, Jana; Sukkar, Iva; Delafuente, Javier; Valcek, Adam; Medvecky, Matej; Jamborova, Ivana; Bitar, Ibrahim; Phan, Minh-Duy; San Millan, Alvaro; Dolejska, Monika	10.1093/jac/dkac250	2022	Europe	Czech Republic
**S007**	Impact of blaNDM-1 on fitness and pathogenicity of Escherichia coli and Klebsiella pneumoniae.	Göttig, Stephan; Riedel-Christ, Sara; Saleh, Ahmad; Kempf, Volkhard A. J.; Hamprecht, Axel	10.1016/j.ijantimicag.2016.02.019	2016	Europe	Germany

**S008**	Decreased Fitness and Virulence in ST10 Escherichia coli Harboring bla(NDM-5) and mcr-1 against a ST4981 Strain with bla(NDM-5).	Zhang, Yawei; Liao, Kang; Gao, Hua; Wang, Qi; Wang, Xiaojuan; Li, Henan; Wang, Ruobing; Wang, Hui	10.3389/fcimb.2017.00242	2017	Asia	China
**S009**	Characterization of Extended-Spectrum Cephalosporin (ESC) Resistance in Salmonella Isolated from Chicken and Identification of High Frequency Transfer of bla(CMY-2) Gene Harboring Plasmid In Vitro and In Vivo.	Kwon, Bo-Ram; Wei, Bai; Cha, Se-Yeoun; Shang, Ke; Zhang, Jun-Feng; Jang, Hyung-Kwan; Kang, Min	10.3390/ani11061778	2021	Asia	Korea
**S010**	Genome-Based Analyses of Fitness Effects and Compensatory Changes Associated with Acquisition of bla (CMY)-, bla (CTX-M)-, and bla (OXA-48/VIM-1)-Containing Plasmids in Escherichia coli.	Pietsch, Michael; Pfeifer, Yvonne; Fuchs, Stephan; Werner, Guido	10.3390/antibiotics10010090	2021	Europe	Germany
**S011**	OXA-48-Mediated Ceftazidime-Avibactam Resistance Is Associated with Evolutionary Trade-Offs.	Fröhlich, Christopher; Sørum, Vidar; Thomassen, Ane Molden; Johnsen, Pål Jarle; Leiros, Hanna-Kirsti S.; Samuelsen, Ørjan	10.1128/mSphere.00024-19	2019	Europe	Norway
**S012**	Colonization of gut microbiota by plasmid-carrying bacteria is facilitated by evolutionary adaptation to antibiotic treatment.	Zhang, Peng; Mao, Daqing; Gao, Huihui; Zheng, Liyang; Chen, Zeyou; Gao, Yuting; Duan, Yitao; Guo, Jianhua; Luo, Yi; Ren, Hongqiang	10.1038/s41396-021-01171-x	2022	Asia	China
**S013**	Effect of plasmids harbouring blaCTX-M on the virulence and fitness of Escherichia coli ST131 isolates.	Shin, Juyoun; Ko, Kwan Soo	10.1016/j.ijantimicag.2015.04.012	2015	Asia	South Korea
**S014**	Porin deficiency or plasmid copy number increase mediated carbapenem-resistant Escherichia coli resistance evolution.	Xiang, Guoxiu; Zhao, Zhiwei; Zhang, Shebin; Cai, Yimei; He, Yuting; Zeng, Jianming; Chen, Cha; Huang, Bin	10.1080/22221751.2024.2352432	2024	Asia	China
**S015**	Piggybacking on Niche Adaptation Improves the Maintenance of Multidrug-Resistance Plasmids.	Kloos, Julia; Gama, João A.; Hegstad, Joachim; Samuelsen, Ørjan; Johnsen, Pål J.	10.1093/molbev/msab091	2021	Europe	Norway

**S016**	The Role of Antibiotic Resistance Genes in the Fitness Cost of Multiresistance Plasmids.	Rajer, Fredrika; Sandegren, Linus	10.1128/mbio.03552-21	2022	Europe	Sweden
**S017**	Occurrence and Biological Cost of mcr-1-Carrying Plasmids Co-harbouring Beta-Lactamase Resistance Genes in Zoonotic Pathogens from Intensive Animal Production.	Lima, Tiago; Loureiro, Dina; Henriques, Ana; Ramos, Fernando; Pomba, Constança; Domingues, Sara; da Silva, Gabriela Jorge	10.3390/antibiotics11101356	2022	Europe	Portugal
**S018**	Co-introduction of plasmids harbouring the carbapenemase genes, bla(NDM-1) and bla(OXA-232), increases fitness and virulence of bacterial host.	Lee, Haejeong; Shin, Juyoun; Chung, Yeun-Jun; Park, Myungseo; Kang, Kyeong Jin; Baek, Jin Yang; Shin, Dongwoo; Chung, Doo Ryeon; Peck, Kyong Ran; Song, Jae-Hoon; Ko, Kwan Soo	10.1186/s12929-019-0603-0	2020	Asia	Korea
**S019**	Hijacking a small plasmid to confer high-level resistance to aztreonam-avibactam and ceftazidime-avibactam.	Ma, Ke; Feng, Yu; McNally, Alan; Zong, Zhiyong	10.1016/j.ijantimicag.2023.106985	2023	Asia	China
**S020**	Pathogenicity of Clinical OXA-48 Isolates and Impact of the OXA-48 IncL Plasmid on Virulence and Bacterial Fitness.	Hamprecht, Axel; Sommer, Julian; Willmann, Matthias; Brender, Christina; Stelzer, Yvonne; Krause, Felix F.; Tsvetkov, Tsvetan; Wild, Florian; Riedel-Christ, Sara; Kutschenreuter, Julia; Imirzalioglu, Can; Gonzaga, Aitor; Nübel, Ulrich; Göttig, Stephan	10.3389/fmicb.2019.02509	2019	Europe	Germany
**S021**	Genomic and functional characterisation of IncX3 plasmids encoding bla(SHV-12) in Escherichia coli from human and animal origin.	Liakopoulos, Apostolos; van der Goot, Jeanet; Bossers, Alex; Betts, Jonathan; Brouwer, Michael S. M.; Kant, Arie; Smith, Hilde; Ceccarelli, Daniela; Mevius, Dik	10.1038/s41598-018-26073-5	2018	Europe	Netherlands
**S022**	Effect of multiple, compatible plasmids on the fitness of the	Lee, Haejeong; Ko, Kwan Soo	10.1093/jac/dkab240	2021	Asia	South Korea

	bacterial host by inducing transcriptional changes.					
**S023**	Characterization of an ST38 carbapenem-resistant and highly virulent Escherichia coli carrying conjugatively transferable ColV virulence-resistance and blaNDM-5-positive resistance plasmids.	Xu, Chen; Li, Yunbing; Li, Yuanyuan; Huang, Lili; Zhou, Mi; Xu, Yi; Dong, Ning	10.1093/jac/dkad403	2024	Asia	China
**S024**	Host dependent maintenance of a bla(NDM-1)-encoding plasmid in clinical Escherichia coli isolates.	Gama, João Alves; Kloos, Julia; Johnsen, Pål J.; Samuelsen, Ørjan	10.1038/s41598-020-66239-8	2020	Europe	Norway
**S025**	Advantage of the F2:A1:B- IncF Pandemic Plasmid over IncC Plasmids in In Vitro Acquisition and Evolution of bla(CTX-M) Gene-Bearing Plasmids in Escherichia coli.	Mahérault, Anne-Claire; Kemble, Harry; Magnan, Mélanie; Gachet, Benoit; Roche, David; Le Nagard, Hervé; Tenaillon, Olivier; Denamur, Erick; Branger, Catherine; Landraud, Luce	10.1128/AAC.01130-19	2019	Europe	France
**S026**	Characterization of NDM-5-positive extensively resistant Escherichia coli isolates from dairy cows.	He, Tao; Wei, Ruicheng; Zhang, Lili; Sun, Lichang; Pang, Maoda; Wang, Ran; Wang, Yang	10.1016/j.vetmic.2017.06.010	2017	Asia	China
**S027**	Interplay between pathogenicity island carriage, resistance profile and plasmid acquisition in uropathogenic Escherichia coli.	Calhau, Vera; Domingues, Sara; Ribeiro, Graça; Mendonça, Nuno; Da Silva, Gabriela Jorge	10.1099/jmm.0.000104	2015	Europe	Portugal
**S028**	Cointegration as a mechanism for the evolution of a KPC-producing multidrug resistance plasmid in Proteus mirabilis.	Hua, Xiaoting; Zhang, Linyue; Moran, Robert A.; Xu, Qingye; Sun, Long; van Schaik, Willem; Yu, Yunsong	10.1080/22221751.2020.1773322	2020	Asia	China
**S029**	Genomic insights into the emergence and spread of NDM-1-producing Vibrio spp. isolates in China.	Zheng, Zhiwei; Xu, Yating; Ye, Lianwei; Chan, Edward Wai Chi; Chen, Sheng	10.1093/jac/dkac276	2022	Asia	China
**S030**	A Novel CMY Variant Confers Transferable High-Level Resistance to Ceftazidime-Avibactam in	Zhou, Junxin; Wang, Weiping; Liang, Min; Yu, Qian; Cai, Shiqi; Lei, Tailong; Jiang, Yan; Du, Xiaoxing;	10.1128/spectrum.03349-22	2023	Asia	China

	Multidrug-Resistant Escherichia coli.	Zhou, Zhihui; Yu, Yunsong				
**S031**	Evolutionary Responses to Acquiring a Multidrug Resistance Plasmid Are Dominated by Metabolic Functions across Diverse Escherichia coli Lineages.	Carrilero, Laura; Dunn, Steven J.; Moran, Robert A.; McNally, Alan; Brockhurst, Michael A.	10.1128/msystems.00713-22	2023	Europe	United Kingdom
**S032**	Characteristics of Plasmids Coharboring 16S rRNA Methylases, CTX-M, and Virulence Factors in Escherichia coli and Klebsiella pneumoniae Isolates from Chickens in China.	Yang, Yongqiang; Zhang, Anyun; Lei, Changwei; Wang, Hongning; Guan, Zhongbin; Xu, Changwen; Liu, Bihui; Zhang, Dongdong; Li, Qingzhou; Jiang, Wei; Pan, Yun; Yang, Chunmei	10.1089/fpd.2015.2025	2015	Asia	China
**S033**	Low biological cost of carbapenemase-encoding plasmids following transfer from Klebsiella pneumoniae to Escherichia coli.	Di Luca, Maria Chiara; Sørum, Vidar; Starikova, Irina; Kloos, Julia; Hülter, Nils; Naseer, Umaer; Johnsen, Pål J.; Samuelsen, Ørjan	10.1093/jac/dkw350	2017	Europe	Norway
**S034**	Molecular Mechanisms Driving the In Vivo Development of KPC-71-Mediated Resistance to Ceftazidime-Avibactam during Treatment of Carbapenem-Resistant Klebsiella pneumoniae Infections.	Li, Xi; Ke, Huanhuan; Wu, Wenhao; Tu, Yuexing; Zhou, Hua; Yu, Yunsong	10.1128/mSphere.00859-21	2021	Asia	China
**S035**	Multidrug resistance plasmids commonly reprogram the expression of metabolic genes in Escherichia coli.	Hall, Rebecca J.; Snaith, Ann E.; Thomas, Matthew J. N.; Brockhurst, Michael A.; McNally, Alan	10.1128/msystems.01193-23	2024	Europe	United Kingdom
**S036**	Persistence of transferable extended-spectrum-β-lactamase resistance in the absence of antibiotic pressure.	Cottell, Jennifer L.; Webber, Mark A.; Piddock, Laura J. V.	10.1128/AAC.00848-12	2012	Europe	United Kingdom
**S037**	Transfer of an Escherichia coli ST131 multiresistance cassette has created a Klebsiella pneumoniae-specific plasmid associated with a major	Sandegren, Linus; Linkevicius, Marius; Lytsy, Birgitta; Melhus, Åsa; Andersson, Dan I.	10.1093/jac/dkr405	2012	Europe	Sweden

	nosocomial outbreak.					
**S038**	Selection pressure required for long-term persistence of blaCMY-2-positive IncA/C plasmids.	Subbiah, Murugan; Top, Eva M.; Shah, Devendra H.; Call, Douglas R.	10.1128/AEM.02788-10	2011	America	United States
**S039**	Characterization of pHeBE7, an IncFII-type virulence-resistance plasmid carrying bla(CTX-M-98b), bla(TEM-1,) and rmtB genes, detected in Escherichia coli from a chicken isolate in China.	Yang, Yan-Xian; Yang, Yong-Qiang; Lei, Chang-Wei; Liu, Bi-Hui; Jiang, Wei; Wang, Hong-Ning; Gazitúa, Maria Consuelo; Zhang, An-Yun	10.1016/j.plasmid.2017.07.001	2017	Asia	China
**S040**	Multicopy plasmids allow bacteria to escape from fitness trade-offs during evolutionary innovation.	Rodriguez-Beltran, Jeronimo; Hernandez-Beltran, J. Carlos R.; DelaFuente, Javier; Escudero, Jose A.; Fuentes-Hernandez, Ayari; MacLean, R. Craig; Peña-Miller, Rafael; San Millan, Alvaro	10.1038/s41559-018-0529-z	2018	America	Mexico
**S041**	An antiplasmid system drives antibiotic resistance gene integration in carbapenemase-producing Escherichia coli lineages.	Zongo, Pengdbamba Dieudonné; Cabanel, Nicolas; Royer, Guilhem; Depardieu, Florence; Hartmann, Alain; Naas, Thierry; Glaser, Philippe; Rosinski-Chupin, Isabelle	10.1038/s41467-024-48219-y	2024	Europe	France
**S042**	Social behaviour involving drug resistance: the role of initial density, initial frequency and population structure in shaping the effect of antibiotic resistance as a public good.	Domingues, I. L.; Gama, J. A.; Carvalho, L. M.; Dionisio, F.	10.1038/hdy.2017.33	2017	Europe	Portugal
**S043**	Live to cheat another day: bacterial dormancy facilitates the social exploitation of β-lactamases	Medaney, F; Dimitriu, T; Ellis, RJ; Raymond, B	10.1038/ismej.2015.154	2016	Europe	United Kingdom
**S044**	The distribution of fitness effects of plasmid pOXA-48 in clinical enterobacteria	Fernández-Calvet, A.; Toribio-Celestino, L.; Del-Valle, A.A.; Sastre-Dominguez, J.; Valdes-Chiara, P.; Millan, A.S.; Delafuente, J.	10.1099/mic.0.001369	2023	Europe	Spain

**S045**	The prevalence of colistin resistance in Escherichia coli and Klebsiella pneumoniae isolated from food animals in China: coexistence of mcr-1 and bla(NDM) with low fitness cost.	Wang, Ruobing; Liu, Yuqing; Zhang, Qing; Jin, Longyang; Wang, Qi; Zhang, Yawei; Wang, Xiaojuan; Hu, Ming; Li, Lulu; Qi, Jing; Luo, Yanbo; Wang, Hui	10.1016/j.ijantimicag.2018.01.023	2018	Asia	China

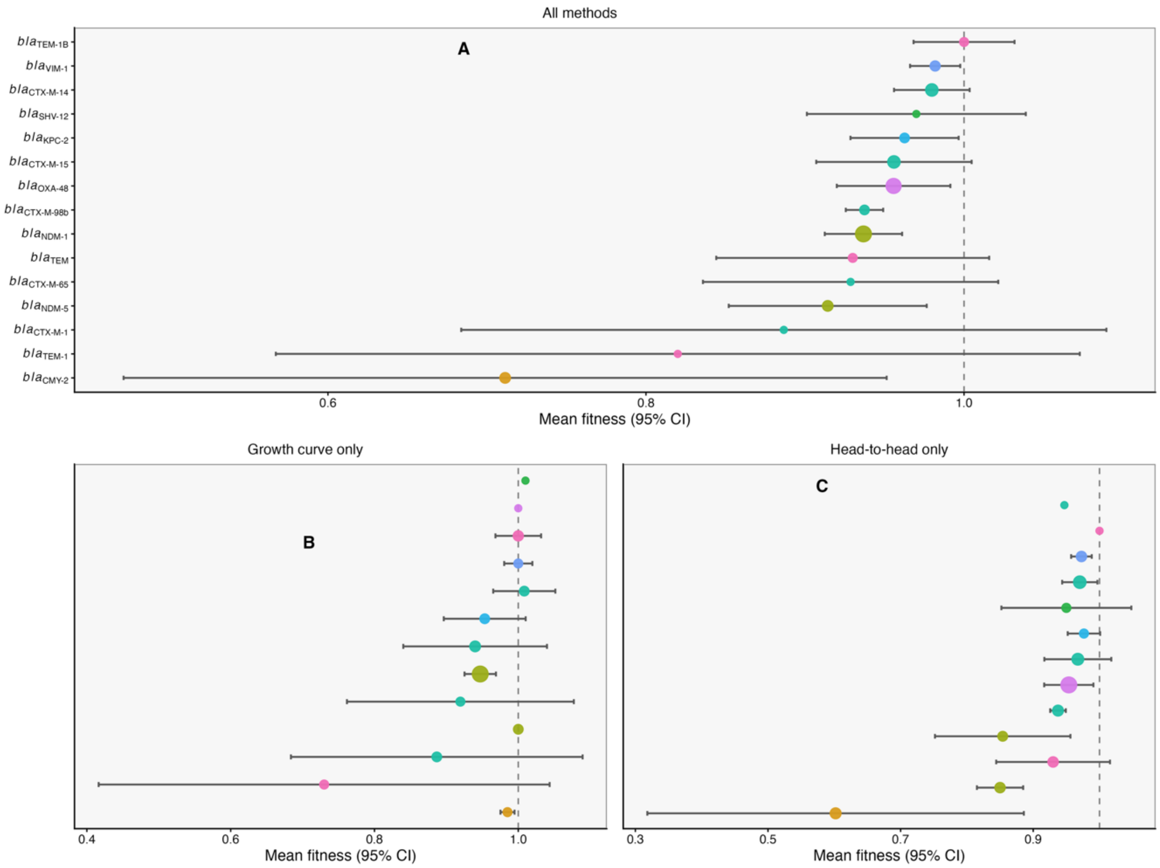

Supplementary Figure S2. Variant-level summaries of standardized fitness estimates. Mean standardized fitness values (w) and 95% confidence intervals are shown for resistance gene variants where variant annotations were available. Point size is proportional to the number of observations contributing to each estimate. The dashed reference line indicates neutrality (w = 1). Because many variant categories are sparsely represented and become further fragmented after stratification by assay type, this figure is intended as descriptive and hypothesis-generating only.
